# Post-Earthquake PTSD and the Role of Telepsychiatry: A Six-Month Follow-Up Study After the 2023 Kahramanmaraş Earthquakes

**DOI:** 10.3390/medicina61061097

**Published:** 2025-06-17

**Authors:** Aila Gareayaghi, Elif Tatlıdil, Ezgi Şişman, Aslıhan Polat

**Affiliations:** 1 Department of Psychiatry, Kocaeli University Research and Application Hospital, 41001 Kabaoğlu, Turkey; elif.tatlidil@kocaeli.edu.tr (E.T.); aslihan.polat@kocaeli.edu.tr (A.P.); 2 Department of Psychiatry, Kocaeli City Hospital, 41060 İzmit, Turkey; drezgisisman@gmail.com

**Keywords:** posttraumatic stress disorder, telepsychiatry, earthquake, disaster psychiatry, depression, Türkiye, trauma, digital mental health

## Abstract

*Background and Objectives:* On 6 February 2023, two catastrophic earthquakes struck southeastern Türkiye, affecting over 13 million individuals and causing widespread destruction. While the physical damage was immediate, the psychological consequences—particularly posttraumatic stress disorder (PTSD) and depression—have proven long-lasting. This study aimed to evaluate the severity and course of PTSD symptoms among survivors and to examine the effectiveness of a telepsychiatry-based mental health intervention in a post-disaster setting. *Materials and Methods:* This naturalistic, observational study included 153 adult participants from the affected regions who underwent at least two telepsychiatry sessions between the first and sixth month post-disaster. Initial screening was conducted using the General Health Questionnaire (GHQ-12), and individuals scoring ≥ 13 were further assessed with the PTSD Checklist—Civilian Version (PCL-C) and the Beck Depression Inventory (BDI). Follow-up evaluations and pharmacological or psychoeducational interventions were offered as clinically indicated. *Results:* At the one-month follow-up, 94.4% of participants met the threshold for PTSD symptoms (PCL-C > 22) and 77.6% had severe depressive symptoms (BDI > 30). By the sixth month, PTSD symptoms had significantly decreased (mean PCL-C score reduced from 42.47 ± 12.22 to 33.02 ± 12.23, *p* < 0.001). Greater symptom reduction was associated with higher educational attainment and perceived social support, while prior trauma predicted poorer outcomes. Depression severity emerged as the strongest predictor of chronic PTSD. *Conclusions:* This study highlights the psychological burden following the 2023 earthquakes in Türkiye and demonstrates the feasibility and potential effectiveness of telepsychiatry in disaster mental health care. Integrating digital mental health services into disaster response systems may help reach vulnerable populations and improve long-term psychological recovery.

## 1. Introduction

Natural disasters have profoundly impacted human physical and psychological well-being throughout history. Türkiye, a country prone to natural disasters and earthquakes, has faced significant devastation over the years and continues to cope with the long-term effects of these events on individuals. Earthquakes in particular stand out as one of the most traumatic natural disasters due to their sudden and unpredictable nature, leading to widespread destruction and loss of life. Large-scale earthquakes can have long-lasting psychological effects on affected individuals, with posttraumatic stress disorder (PTSD) one of the most prominent outcomes resulting from intense stress and traumatic experiences [1].

On 6 February 2023, two powerful earthquakes with magnitudes of Mw 7.7 and Mw 7.6 struck southeastern Türkiye within nine hours of each other, unleashing one of the deadliest natural disasters in the country’s modern history. The tremors affected an area spanning over 110,000 square kilometers and directly impacted more than 13 million people across 11 provinces. These earthquakes caused unprecedented destruction across the region, resulting in over 50,000 confirmed deaths and hundreds of thousands of injuries [2]. Thousands of buildings—including apartment blocks, hospitals, and schools—collapsed instantly, while critical infrastructure such as roads and communication networks were rendered inoperable. The initial hours were marked by chaos and silence: countless individuals lay trapped under debris, many of them still alive, their voices fading as time passed. For survivors, the horror was not only the destruction but also the unbearable helplessness of hearing loved ones and strangers cry out from beneath the rubble—calls that would go unanswered.

In the days following the earthquake, temperatures dropped below freezing. Snowfall and biting cold added a cruel dimension to the tragedy, as displaced families gathered around improvised fires in open fields without electricity, heating, or clean water. Tens of thousands were forced to spend nights in cars, under tarps, or in hastily erected tents with little protection against the harsh winter. The delivery of aid was hampered by damaged roads and overwhelmed systems, and in some regions, help did not arrive for days. Bodies recovered from the rubble had to be laid to rest swiftly in mass graves due to health concerns, often without proper identification or religious rituals. In many cities, the scent of concrete dust and death lingered in the air for weeks.

As the acute phase gave way to prolonged crisis, hundreds of thousands of survivors were relocated to tent cities and container settlements, where some of them continue to live more than two years later. Aftershocks—some exceeding magnitude 5.0—persisted for months, sustaining a climate of chronic threat and reactivating trauma responses. The loss of homes, family members, routines, and a sense of safety have left deep psychological scars. Children who witnessed the collapse of their homes, adults who buried loved ones without closure, and entire communities that vanished in minutes now struggle with an invisible aftermath: grief, guilt, and despair. Under such conditions, mental health is not a secondary concern—it is a cornerstone of recovery.

The impact of such disasters is not limited to the general population—health-care workers are also significantly affected. These professionals not only experience the trauma firsthand but also bear the responsibility of providing essential medical services under extreme conditions. In natural disasters like these, health-care workers form the backbone of survival support and disaster management. However, many health-care workers lost their homes, family members, and even their lives, while health-care facilities also suffered extensive damage, rendering some of them inoperable. The earthquakes forced many health-care workers to leave the region, creating a severe shortage in medical services. According to data from the Turkish Medical Association, 463 health-care workers, including 107 physicians, lost their lives in the earthquake-affected regions, while 6 health-care workers, including 5 physicians, were reported missing [3]. To address this gap, from the first day of the disaster, 21,204 physicians and 62,590 health-care workers were deployed to the affected areas on five-day shifts to provide medical assistance and support recovery efforts [4]. However, ensuring the continuity of mental health services and maintaining established therapeutic relationships in the long term required innovative solutions.

One of the authors of this study was directly involved in post-earthquake relief efforts, and upon returning, observed the severe shortage of mental health professionals in the affected areas. Access to mental health support through specialized organizations was limited due to challenges in physically reaching the affected population, a shortage of qualified health-care professionals, financial constraints, and the social stigma connected with mental health care. As of then, digital mental health interventions had not been implemented in Türkiye’s public or university hospitals except from our clinic. Recognizing the urgent need for continued psychiatric support, our clinic answered with an immediate digital mental health-care response, offering remote consultations to earthquake survivors. This experience highlighted the critical role of telepsychiatry, not only in PTSD assessment but also in the management of preexisting chronic psychiatric conditions.

In recent years, technological advancements have facilitated the expansion of telemedicine and telepsychiatry, making mental health support more accessible to larger populations in the aftermath of disasters. Regardless of whether a disaster occurs, health cannot be discussed without considering mental health. When physical access to health-care services is disrupted, telepsychiatry serves as a vital tool for ensuring continued mental health support and recovery [5]. Tele-mental health interventions were found to play a key role in delivering trauma-informed care to populations affected by natural disasters [6]. Following the Kahramanmaraş earthquakes, the demand for telepsychiatry services increased significantly, demonstrating their effectiveness in enhancing psychological well-being.

The hospital conducting this study has been utilizing telepsychiatry since the COVID-19 pandemic, during which our clinic also conducted group therapy sessions through telepsychiatry [7]. In accordance with the Ministry of Health’s guidelines [8], we also utilize telepsychiatry services to monitor postpartum depression in new mothers. Also, because we are one of the few gender dysphoria clinics operating in Türkiye, we find that telepsychiatry serves a great tool to follow up patients experiencing gender dysphoria who face challenges in accessing hospital-based care. These experiences have proven quite valuable, extending telepsychiatric services beyond disaster victims to individuals in remote locations with limited access to mental health care. Our unit swiftly utilized its existing telepsychiatry infrastructure by establishing a disaster clinic within the first week of the earthquake.

This study aimed to examine the sociodemographic factors associated with PTSD development, assess traumatic stress symptoms, and evaluate the effectiveness of telepsychiatry services and psychiatric medication following the February 6 earthquakes. By gaining deeper insights into the psychiatric impact of traumatic experiences on earthquake survivors and optimizing psychosocial support systems, we sought to study the effectiveness of telepsychiatry services in order to enhance disaster mental health services for the future. This research highlights the importance of providing psychoeducation and self-care information to affected individuals in the region. Additionally, this study highlights the relevance of digital approaches for mental health follow-up in disaster settings, in line with recent meta-reviews on the efficacy of web-based interventions for trauma-related conditions [9].

## 2. Methods

### 2.1. Study Design

This was a naturalistic, observational study carried out with patients from the regions directly affected by the 6 February 2023 earthquakes in Türkiye. The primary aim was to assess psychological distress, provide supportive interventions, and evaluate posttraumatic stress disorder (PTSD) symptoms over time. The study involved online video consultations with affected individuals, integrating both clinical assessments and psychoeducation, with pharmacological interventions when necessary.

During the first month following the earthquakes, a total of 211 individuals were interviewed and screened using the General Health Questionnaire (GHQ-12). Of these, 189 participants scored 13 or higher and were classified as being at psychological risk. Psychoeducational recommendations were provided to all, and those who scheduled follow-up appointments and continued to reside in the disaster-affected area (n = 156) were assessed at the end of the first month using the PTSD Checklist—Civilian Version (PCL-C) and the Beck Depression Inventory (BDI). Three individuals were excluded due to acute psychiatric conditions—two experiencing psychotic episodes and one presenting with delirium—resulting in a final study sample of 153 participants, each of whom completed at least two consultations. Although PCL-C assessments were initially planned to be conducted every three months, the extraordinary circumstances following the disaster made this unfeasible, and follow-up was ultimately conducted at the six-month mark. At that time, 102 participants were already under ongoing psychiatric follow-up at variable intervals. Among the 51 individuals who had dropped out, 10 could not be reached, but follow-up evaluations were successfully conducted with the remaining 41. Thus, a total of 143 participants completed the six-month assessment.

### 2.2. Ethics Approval

Ethics approval for the study was obtained from the Kocaeli University Clinical Research Ethics Committee (approval KÜ GOKAEK-2023/13.37, project 2023/261). The research was conducted in compliance with the Declaration of Helsinki, ensuring ethical principles in medical research involving human subjects. Written informed consent was obtained from all participants before their participation in the study.

### 2.3. Participants and Recruitment

A total of 143 individuals affected by the earthquake completed the study. The mean age of the participants was 38.01 ± 13.75 years, with 79.7% (n = 114) identifying as female and 20.3% (n = 29) as male.

Recruitment was conducted through multiple channels, including collaboration with the Psychiatric Association of Türkiye, direct engagement by field teams providing face-to-face support in the affected region, targeted social media announcements, and outreach via family physicians working in the region, many of whom were alumni of our institution.

Participants were eligible for inclusion if they were 18 years of age or older and had been directly affected by the earthquake. To ensure greater clarity and methodological rigor, exclusion criteria included the presence of an acute psychiatric episode, individuals with significant cognitive impairment, active substance use disorder, and inability to access digital platforms at the time of initial assessment. Three participants were excluded from the study: one patient experiencing a schizophrenic episode, another experiencing a manic episode, and a third exhibiting delirium related to dementia.

### 2.4. Data Collection and Assessment Procedures

Appointments were scheduled through the hospital’s official appointment system, ensuring structured access to the consultations. All video interviews were conducted remotely via videoconference. Apart from our previous telepsychiatry practices, since the patients were still in the disaster or living in tent cities, they were connected to the system through their cell phones instead of computers at the time of scheduling. Each session lasted approximately 30 min and was conducted during working hours.

During the initial consultation, participants were asked to complete a set of standardized self-report scales: the Posttraumatic Stress Disorder Checklist—Civilian Version (PCL-C), the Beck Depression Inventory (BDI), and the General Health Questionnaire (GHQ). In addition, demographic data—including age, gender, occupation, marital status, and psychiatric history—were collected during the video consultations. The interviewer also asked patients a series of questions about their earthquake experience and recorded their complaints in an open-ended manner.

### 2.5. General Health Questionnaire—12 Items (GHQ-12)

The GHQ-12 is a brief screening tool developed to detect general psychological distress in community and clinical settings. The scale comprises 12 items scored using a Likert method (0–1–2–3), with higher scores indicating greater levels of distress. In this study, a cut-off score of 13 was used to identify individuals at psychological risk, as previously validated in the Turkish population [10]. The GHQ-12 has been widely utilized in post-disaster mental health research due to its brevity and sensitivity.

### 2.6. Posttraumatic Stress Disorder Checklist—Civilian Version (PCL-C)

The PTSD Checklist—Civilian Version (PCL-C) is a 17-item self-report instrument developed to assess the presence and severity of posttraumatic stress disorder (PTSD) symptoms in individuals exposed to traumatic events. Items correspond to the DSM-IV criteria for PTSD and are grouped into three subscales: re-experiencing (5 items), avoidance/numbing (7 items), and hyperarousal (5 items). Responses are rated on a 5-point Likert scale ranging from 1 (“not at all”) to 5 (“extremely”), with higher scores indicating greater symptom severity. The total score ranges from 17 to 85. In the Turkish adaptation of the scale, a cut-off score between 22 and 24 has been shown to offer optimal sensitivity and specificity (sensitivity > 72%, specificity > 80%) for identifying PTSD [11]. In our study, we used a cut-off score of 23 to determine clinically significant PTSD symptoms. The PCL-C was administered at baseline (1st month) and at follow-up (6th month) to assess changes in PTSD symptom severity. The scale has demonstrated high internal consistency in Turkish populations (Cronbach’s α = 0.922) and is a valid and reliable instrument for use in post-disaster mental health evaluations [11].

### 2.7. Beck Depression Inventory (BDI)

The Beck Depression Inventory (BDI) is a self-report scale developed by Aaron T. Beck in 1961 to evaluate the presence and severity of depressive symptoms across emotional, cognitive, somatic, and motivational domains. It is one of the most widely used instruments in both clinical practice and research settings. The BDI consists of 21 items, each corresponding to a specific symptom of depression. Among these, 2 items assess affective symptoms, 11 items assess cognitive symptoms, 2 items address behavioral aspects, 5 items evaluate somatic complaints, and 1 item reflects interpersonal difficulties. Each item is rated on a 4-point Likert scale ranging from 0 to 3, with higher scores indicating more severe symptomatology. In our study, a cut-off score of 17 was used to indicate clinically significant depressive symptoms, in line with previous studies conducted in Turkish populations. Based on the total score, depression severity is categorized as 0–9 (no depression), 10–16 (mild), 17–23 (moderate), and ≥24 (severe depression). The Turkish version of the BDI has been previously validated and demonstrated satisfactory psychometric properties in clinical and non-clinical populations [12].

### 2.8. Follow-Up and Interventions

Following the initial assessment, participants were scheduled for follow-up sessions at variable intervals ranging from biweekly to monthly, depending on their clinical needs and availability. During these sessions, supportive psychotherapy was provided, and pharmacological treatment was initiated when clinically necessary. The prescribed medications included selective serotonin reuptake inhibitors (SSRIs), serotonin–norepinephrine reuptake inhibitors (SNRIs), and sleep medications. Importantly, benzodiazepines were not prescribed as part of the treatment protocol. There was no standardized dosing regimen, as all pharmacological treatments were tailored to individual patient needs based on symptom severity and clinical response. This follow-up strategy was in line with recent evidence supporting the use of personalized, digital CBT approaches to improve continuity and adherence in post-crisis psychiatric care [13].

To assess changes in PTSD symptoms over time, participants were asked to complete the PCL-C again six months after their initial consultation. This follow-up assessment was used to reevaluate PTSD diagnoses and monitor symptom progression.

### 2.9. Statistical Analysis

Data analysis was conducted using SPSS V.22.0 (Statistical Package for Social Sciences) for Windows. Paired *t*-tests were applied for comparing dependent variables in pairwise analyses. Pearson correlation coefficients were calculated to assess the relationships between continuous variables. To explore higher-order effects and potential predictors, multiple regression analyses were performed. Statistical significance was determined at an alpha level of 0.05 (α = 0.05).

## 3. Results

### 3.1. Sociodemographic Characteristics of Participants

Out of 146 applicants, 143 were included in the analysis. The sample was predominantly female (79.7%), with a mean age of 38.01 years (SD = 13.75). Educational attainment was relatively high: over half (57.3%) had at least a university degree, suggesting a potentially resourceful population. However, employment status revealed vulnerability—only 46.9% were employed post-disaster.

Family dynamics also provided insight into caregiving burdens, with participants reporting an average of 1.5 children (SD = 1.63). Additionally, 42% had caregiving responsibilities for a dependent individual, indicating that a significant portion were managing both personal trauma and the well-being of others.

All the participants were still residing there despite disaster impacts. Hatay, one of the hardest-hit provinces, accounted for the largest regional subgroup (35.7%).

Lifestyle data painted a picture of increased distress: 31.5% of participants were smokers, and among them, 60% reported increased smoking after the earthquake, a likely indicator of heightened stress or deteriorating coping mechanisms. Additionally, 9.1% consumed alcohol and nearly one in four (23.8%) had at least one chronic medical condition, potentially compounding vulnerability (Table 1).

### 3.2. Disaster Impact and Access to Resources

The impact of the earthquake on daily life was substantial. Over 60% of participants reported direct personal losses: 51.7% lost their homes, 23.8% experienced property damage, and 18.2% encountered disruptions in their work, income, or education. While most participants (93.7%) reported having access to some form of financial support—predominantly through personal savings or assistance from family and friends—nearly 7 out of 10 (68.5%) encountered significant difficulties in accessing formal aid or relief services.

Regarding mental health services, 44.1% of participants had received psychiatric treatment prior to the earthquake, while 23.1% sought professional help afterward. Strikingly, all the participants reported psychiatric symptoms following the disaster, underscoring the widespread and ongoing psychological distress experienced by survivors (Table 2**)**.

### 3.3. PTSD, Depression, and Trauma Exposure

Physical injuries were reported in 15.4% of participants, with 1.4% sustaining severe injuries and admitted to the hospital. The 2.8% with moderate injuries received outpatient medical treatment. Additionally, 42% of participants reported being responsible for the care of a dependent individual, 93.7% reported having social support, and 14.7% indicated participation in volunteer rescue team efforts.

Participants’ prior trauma experiences revealed that 31.5% had encountered significant personal hardships, such as the loss of a loved one, divorce, serious illness, legal issues, job loss, or extreme poverty. Furthermore, 81.8% reported fearing for their own life during the earthquake, and 86% feared for the lives of their loved ones. Additionally, 30.8% witnessed a relative being injured or dying, and 45.5% learned of a loved one’s death from another family member. Among those who lost a relative, 36.4% reported that an appropriate funeral ceremony could not be conducted, and 33.6% of the participants could not even find the bodies of their lost (Table 2 and Table 3).

### 3.4. Psychiatric Symptoms over Time

The GHQ was administered in the first month, and scores above 13 were included in the study. The mean GHQ score of the group was 19.34 ± 2.97.

At the end of the first month, mean PCL-C scores were 42.47 (SD = 12.22) and Beck Depression Inventory scores were 36.39 (SD = 9.69). At the end of the sixth month, mean PCL-C scores were 33.02 (SD = 12.23) (t = 7.242, *p* < 0.001). Significant improvements were observed in the re-experiencing, avoidance, and hyperarousal subscales (*p* < 0.001 for all). PTSD symptoms decreased significantly over the six-month follow-up period (Table 4).

PCL-C scores were moderately correlated with depressive symptoms (BDI: r = 0.451, *p* < 0.001).

### 3.5. Predictors of Symptom Improvement and Chronic PTSD

Regression analyses revealed that three key factors significantly predicted greater symptom improvement over time. Higher educational attainment (*p* = 0.029) and access to social support (*p* = 0.027) were both associated with stronger recovery trajectories. Conversely, participants with a history of traumatic experiences showed less symptom reduction (*p* = 0.029), indicating that cumulative trauma may impair psychological resilience (Table 5).

At the six-month follow-up, chronic PTSD was significantly associated with higher BDI scores (*p* = 0.018), while GHQ scores, age, gender, and perceived social support were not significant predictors (Table 6).

## 4. Discussion

This study employed a naturalistic, observational design to investigate the psychological impact of the 6 February 2023 earthquakes in Türkiye through a telepsychiatry-based intervention. A total of 211 individuals were initially screened via the General Health Questionnaire (GHQ-12), and 189 participants scoring ≥ 13 were considered at psychological risk. Of these, 156 individuals residing in the disaster area and opting for follow-up were assessed with the PTSD Checklist—Civilian Version (PCL-C) and the Beck Depression Inventory (BDI) at the end of the first month. After excluding three individuals due to acute psychiatric conditions, the final sample included 153 participants. PCL-C scores showed a significant decline from a baseline mean of 42.47 (SD = 12.22) to 33.02 (SD = 12.23) at the six-month follow-up, indicating substantial symptom improvement. All PCL-C subscales—re-experiencing, avoidance, and hyperarousal—demonstrated significant reductions. Additionally, BDI scores at baseline were high (mean = 36.39, SD = 9.69), and regression analysis revealed that greater PTSD symptom reduction was associated with higher education and perceived social support, while trauma history predicted poorer outcomes. Of the initial cohort, 143 participants completed the six-month follow-up, validating the feasibility and effectiveness of telepsychiatry in delivering post-disaster mental health care under extraordinary conditions.

In this study, we observed that women were more likely to experience persistent posttraumatic symptoms—findings that align with prior post-disaster research. Zhou et al. identified female gender, older age, and solitary living as predictors of PTSD symptom persistence six months after the Wenchuan earthquake [14]. The influence of gender on posttraumatic stress responses is strongly supported in the literature. Numerous studies have demonstrated that women are more vulnerable to developing PTSD compared to men. A review by Farooqui et al. (2017) [15] encompassing 77 earthquake studies and a meta-analysis by Nemeroff et al. (2006) [16] both revealed that women were nearly twice as likely as men to develop PTSD. Field research conducted after the 2023 Türkiye Kahramanmaraş earthquakes also confirmed this finding, reporting a PTSD prevalence in women that was approximately double that of men [17]. Similarly, analyses of earthquakes in Iran, Pakistan, and Haiti have identified female gender as a significant risk factor [18,19]. After the 2011 earthquake in Japan, older women were also found to have a 1.6-fold higher prevalence of PTSD symptoms compared to men [20]. These findings across diverse populations suggest that women may be more susceptible to trauma due to biological and psychosocial mechanisms. Heightened sensitivity to stress hormones, a tendency to interpret trauma as more threatening, and the burden of traditional gender roles are among the proposed contributors to this increased risk [21,22]. Our predominantly female sample underscores the need to consider gender-specific risk factors. In our experience, women survivors of the earthquake often faced unique stressors that exacerbated their mental health symptoms. Several participants reported intense psychological distress stemming from the lack of private sanitation facilities, limited access to menstrual hygiene products, and the burden of sharing cramped tent or container spaces with their spouses—particularly among those experiencing intimate partner violence. These compounded vulnerabilities frequently contributed to the persistence or worsening of depressive and posttraumatic symptoms. Through telepsychiatry, many of these women were able to access confidential support, receive psychoeducation on trauma and coping, and when appropriate, initiate pharmacological treatment. Despite infrastructural limitations, remote care provided a crucial lifeline for these patients, demonstrating the viability and importance of telepsychiatry in addressing gender-specific needs after disasters. In our experience, women may be more prone to intrusive rumination and emotional overprocessing following trauma, which has been shown to mediate PTSD severity [23]. This gendered vulnerability is also supported by findings in adolescents, where female participants consistently exhibited higher levels of depression, anxiety, and PTSD-related symptoms post-earthquake [24]. Digital access also may present an additional gender-related challenge. Although telepsychiatry offers a promising route for care continuity, women in certain conditions may face unique barriers, such as limited digital literacy, cultural stigma, and concerns over data privacy. These factors can hinder engagement with digital mental health services, especially in post-disaster contexts [13].

In our study, lower educational attainment was significantly associated with increased severity of PTSD symptoms. This finding aligns with a substantial body of literature underscoring the impact of education on posttraumatic psychological outcomes. Following the Wenchuan earthquake, large-scale analyses identified low education as a significant risk factor for PTSD development [22,25]. Similarly, in a systematic review by Sirotich and Camisasca (2024) [26], low educational status was highlighted among the primary sociodemographic risk factors for disaster survivors, alongside unemployment and financial hardship. This vulnerability may stem from limited problem-solving skills, reduced access to information and services, and less effective coping strategies among individuals with lower education. These individuals also tend to have weaker social support networks and are more likely to engage in adverse health behaviors [26]. The stronger and more persistent PTSD symptoms observed among participants with lower education in our sample further support this risk profile. Therefore, post-disaster interventions should incorporate educational and socioeconomic indicators into risk assessments to enable early identification and targeted support for high-risk groups.

A history of trauma also emerged as a significant risk factor for more severe post-disaster symptoms. This finding aligns with existing literature suggesting that prior trauma may serve as an additional vulnerability factor in the face of new traumatic events. The literature on complex trauma shows that early-life interpersonal trauma—such as childhood abuse, neglect, or loss—can increase vulnerability to anxiety and trauma-related disturbances in adulthood [27]. Research has shown that previously traumatized individuals are significantly more likely to develop PTSD after a disaster [25,28]. Meta-analyses focusing on post-earthquake mental health consistently identify prior trauma as a strong risk factor for PTSD [22]. Individuals with earlier trauma may experience subsequent disasters as a reactivation of past experiences, thereby intensifying their psychological response [29]. This vulnerability appears to be especially pronounced when the past and current traumas share thematic or contextual similarities. Cumulative traumatic exposures may deplete coping resources and amplify stress reactions in a synergistic manner, while also heightening preexisting anxiety and perceived danger in the world [30]. The persistence of PTSD symptoms among trauma-experienced participants in our sample supports these mechanisms. These findings underscore the importance of systematically screening for trauma history in post-disaster interventions and providing targeted support to individuals with prior traumatic exposure.

Conversely, certain factors appeared to buffer participants against persistent symptoms. Our study found a significant association between lack of social support and the severity of PTSD symptoms, aligning with extensive evidence in the literature emphasizing the critical role of social support in posttraumatic psychological recovery. Numerous studies have shown that strong perceived social support serves as a protective factor against posttraumatic stress, whereas low levels of support are linked to increased PTSD risk [25]. For instance, meta-analyses examining risk factors after natural disasters have demonstrated that adequate social support significantly reduces the likelihood of developing PTSD. The “social support deterioration” model proposed by Kaniasty and Norris (1995) suggests that the gradual erosion of social networks after disasters may impair coping capacities and exacerbate psychological distress [31]. In line with this, research following the 2008 Wenchuan earthquake found that individuals with strong social support had significantly lower PTSD rates [22]. Social support not only facilitates the recovery process but also acts as a buffer against the adverse effects of extreme stress. Although some longitudinal studies indicate that the protective effects of support may diminish over time—or even re-trigger trauma in some contexts—overall, the evidence supports its beneficial role. Strengthening social ties and maintaining support networks in the aftermath of disasters remain essential strategies for both PTSD prevention and psychological resilience [26]. These findings align with studies showing that psychological strengths at the individual and family level—such as strength-based parenting—can promote resilience and even posttraumatic growth, especially when mediated by traits like optimism [32].

The digital delivery of psychiatric care played a crucial role in our intervention model reducing PTSD symptoms over a six-month period. Our findings add to the growing evidence base that guided digital interventions, particularly those involving synchronous therapist contact, can be effective and cost-efficient alternatives to face-to-face therapy in the treatment of PTSD and depression. Synchronous modalities such as video consultations are especially valuable in enhancing therapeutic alliance and treatment adherence compared to asynchronous methods [9]. There is growing evidence in the literature that telepsychiatry can fill critical gaps in care, particularly in regions where infrastructure has been damaged or access to specialists is limited. For instance, Augusterfer et al. (2018) reported that telepsychiatry and digital health technologies have effectively supported post-disaster mental health services in over 85 countries [33]. Similarly, following the 2015 earthquake and floods in Pakistan, a regional telepsychiatry network successfully provided psychiatric care where in-person services were unavailable [34]. Reinhardt et al. (2019) also emphasized that telepsychiatry reduced waiting times, improved access, and was generally well accepted by both patients and clinicians [35]. The symptom improvement observed in our sample provides a real-world example of telepsychiatry’s potential efficacy in managing PTSD. Although the number of controlled studies remains limited, current data suggest that this modality offers significant advantages in terms of service continuity and early intervention [36,37]. Therefore, telepsychiatry should be considered a viable and effective alternative in post-disaster settings where face-to-face care is not feasible. However, it should not be overlooked that access inequities remain a challenge, especially for individuals from marginalized or economically disadvantaged groups who may lack the infrastructure or digital literacy needed to fully engage with these platforms [9]. There is also a larger systemic issue that should be addressed when it comes to the use of telepsychiatry. As Ahmed and Heun emphasize, the absence of internationally standardized guidelines for electronic mental health and psychosocial support in humanitarian settings creates a barrier to quality and equity in service delivery. There is a need for structured frameworks that address not only clinical care but also ethical, logistical, and technological aspects of digital interventions [37].

Several post-disaster telepsychiatry interventions have been reported following major earthquakes, providing a basis for comparison with the current study. Table 7 summarizes key characteristics of telepsychiatry studies after the 2010 Haiti earthquake, the 2008 Wenchuan (China) earthquake, and the 2005 Pakistan earthquake, including each study’s design, population, intervention, and main findings. Notably, prior efforts demonstrated the feasibility of remote mental health care in disaster settings. For example, in post-earthquake rural Haiti (2010), a pilot initiative integrated a visiting U.S. psychiatrist with local providers at five clinics, using the WHO mhGAP protocol to support treatment of common mental disorders [38]. A retrospective review from that Haiti program found that 65 patients were seen (most with depression, epilepsy, or chronic mental illnesses) and 90% required follow-up care. Importantly, very few patients identified the earthquake as the direct cause of their psychiatric symptoms, underscoring substantial preexisting mental health needs [38]. In China (Wenchuan 2008), an innovative approach was a web-based self-help program (My Trauma Recovery) evaluated in an RCT. In that study, trauma survivors who used the internet-delivered intervention had significantly reduced PTSD symptoms compared to controls, with large effect sizes observed after 1 month of use and sustained improvement at 3 months [39,40]. In Pakistan (2005), where mountainous terrain and infrastructure damage limited in-person care, telepsychiatry was implemented to connect specialists with remote field clinics. Reports from Pakistan’s telepsychiatry program describe it as a practical and feasible strategy for delivering psychiatric rehabilitation to displaced survivors in austere settings [40].

In our study, a strong association was found between the severity of posttraumatic depressive symptoms and PTSD symptom levels. Participants with higher Beck Depression Inventory (BDI) scores exhibited more severe and persistent PTSD symptoms. This finding aligns with extensive literature indicating that PTSD and depression frequently co-occur in the aftermath of trauma. For instance, in a study conducted after the 1999 Marmara earthquake, the lifetime prevalence of PTSD and major depressive disorder (MDD) was found to be 19.2% and 18.7%, respectively, and 37.5% of those with PTSD also met diagnostic criteria for depression [41]. Similarly, studies following the 2010 Haiti and 2008 Sichuan earthquakes reported strong correlations between PTSD and depressive symptoms [19,42]. Research has consistently shown that the co-occurrence of these disorders is associated with more severe clinical presentations and poorer recovery outcomes. In our sample, higher BDI scores were predictive of persistent PTSD at the six-month follow-up, suggesting that depression may contribute to the chronicity of PTSD. Therefore, screening for depressive symptoms in post-disaster mental health assessments is essential for identifying individuals at risk for PTSD. As emphasized in previous studies, those who develop depression after trauma are also highly susceptible to PTSD, highlighting the need for integrated psychosocial interventions that address both conditions simultaneously [24].

It has been widely emphasized in the literature that the comorbidity of chronic PTSD and depression is highly prevalent following earthquakes. For instance, after the 1999 Marmara earthquake, the prevalence of PTSD was reported to be 23%, while the rate of comorbid depression was 16% [29]. Similarly, a meta-analysis on the 2010 Haiti earthquake indicated that approximately a quarter of survivors exhibited PTSD symptoms and a third showed depressive symptoms [19]. In certain samples, the rate of comorbidity was even higher: for example, 39.2% of adolescents affected by the 2017 Jiuzhaigou earthquake exhibited both PTSD and depression [43]. The frequent co-occurrence of PTSD and depression can lead to more severe and treatment-resistant symptom trajectories. Indeed, a study conducted after the Haiti earthquake demonstrated a significant positive correlation between PTSD and depressive symptom severity [44]. Depression often develops secondarily in the aftermath of trauma and may contribute to the chronicity of PTSD. In line with this, a study on child survivors of the Lushan earthquake reported that PTSD symptoms typically emerged earlier than depressive symptoms, suggesting that depression might exacerbate preexisting trauma-related distress [44]. Longitudinal studies further support this interaction: for instance, one study showed that while PTSD symptoms significantly decreased 13 months after an earthquake, depression levels—measured by the Beck Depression Inventory (BDI)—remained largely unchanged [45]. This finding highlights that untreated depression may sustain PTSD symptoms over time. Furthermore, higher BDI scores have been associated with more severe PTSD symptoms among earthquake survivors [44]. The findings of our current study are largely consistent with the existing literature: we similarly observed that depression was prevalent among chronic PTSD cases and that higher BDI scores were positively correlated with greater PTSD symptom severity. Although some variability in prevalence rates has been reported across different studies—for instance, comorbidity rates tend to be higher among adolescents compared to adults [43]—there is a general consensus that PTSD and depression comorbidity constitutes a major clinical challenge following earthquakes. Accordingly, the literature emphasizes the critical importance of early identification and comprehensive treatment of both disorders among disaster survivors [45]. Early psychosocial interventions have been suggested to be effective in alleviating PTSD symptoms and preventing the chronicity of PTSD by simultaneously treating comorbid depression [45]. Furthermore, given that psychiatric problems can persist for many years after major earthquakes—for example, PTSD and depression rates remained as high as 11.8% and 24.8%, respectively, even eight years after the Wenchuan earthquake [46]—it is recommended that long-term follow-up and intervention programs be systematically planned.

The phenomenon that PTSD symptoms may spontaneously diminish over time is well documented in the literature. Although the majority of individuals exposed to trauma experience some PTSD symptoms during the first few weeks, these typically subside on their own. It has been reported that only a small proportion of trauma-exposed individuals (approximately 8%) fail to exhibit this “natural recovery” and go on to develop PTSD [47]. Longitudinal studies have shown that there is a marked decrease in PTSD symptoms during the acute posttrauma period, typically within the first 3 to 6 months. For instance, meta-analytic evidence indicates that PTSD prevalence is around 25% within the first month following trauma, but this rate decreases by approximately a third by the third month. Similarly, a systematic review found that nearly half of individuals diagnosed with PTSD achieved remission within three years without receiving any formal treatment [48]. Rates of natural recovery can vary depending on factors such as the type of trauma and the initial symptom profile. Indeed, recovery over time tends to be higher among individuals exposed to single-incident and unintentional traumas (e.g., natural disasters), whereas it is reported to be lower following human-made, intentional traumas [49]. When examining symptom dimensions, individuals exhibiting pronounced hyperarousal symptoms have been found to experience poorer outcomes—namely persistently elevated symptom levels—at 12 months, suggesting that spontaneous recovery is less likely in this subgroup [50]. On the other hand, protective factors such as social support play a critical role in the process of natural recovery: strong social support following trauma has been shown to reduce the risk of developing PTSD and to facilitate the spontaneous resolution of symptoms [47]. These findings have fueled debate over whether immediate, intensive psychological intervention is necessary for every individual exposed to trauma. The prevalence of natural recovery has led clinicians to adopt a “watchful waiting” or active monitoring approach in some cases. In particular, for individuals who exhibit relatively mild symptoms within the first few weeks, it is often recommended to allow time for potential spontaneous recovery before initiating formal treatment [48,51]. Universal early interventions—such as single-session structured psychological debriefing—have not been found effective in preventing chronic PTSD, and in some cases may even disrupt the natural course of recovery. Indeed, it has been suggested that prematurely pathologizing normal acute stress reactions after trauma may undermine individuals’ confidence in their own coping abilities. Accordingly, current clinical guidelines recommend monitoring individuals who exhibit PTSD symptoms within the first month posttrauma through regular follow-up, allowing time for spontaneous recovery. Trauma-focused treatments should be initiated only if symptoms fail to improve significantly over time or become more severe [51]. In conclusion, longitudinal research on the natural course of PTSD has shown that a significant reduction in symptom severity occurs within 6 to 12 months for a substantial proportion of cases. However, a subset of individuals may experience a chronic course lasting for years in the absence of clinical intervention [48,49]. In the present study, the decrease observed in PCL-C scores at the 6-month follow-up aligns with the natural recovery trajectory emphasized in the literature. However, early identification of high-risk individuals who do not exhibit spontaneous recovery is crucial, as timely clinical intervention for these individuals is essential to prevent the long-term development of chronic PTSD [50,51].

Advances in artificial intelligence (AI) are poised to augment telepsychiatry by triaging patients, tracking symptoms, and providing chatbot-based support at scale. During the COVID-19 pandemic, for example, an AI-driven virtual triage system handled millions of online symptom-check encounters and effectively flagged individuals with mental health needs for care referral [52]. Such AI triage tools can rapidly detect early signs of distress in large populations, potentially accelerating access to treatment during disasters. Likewise, AI chatbots are emerging as a scalable means of delivering psychological support. A recent trial of a generative AI “therapy chatbot” found significant reductions in users’ depression and anxiety symptoms—a 51% drop in depressive symptoms on average—comparable to outcomes from traditional therapy. Participants reported trusting the chatbot and engaging with it much like a human therapist [53]. These findings demonstrate the potential for AI-assisted telepsychiatry to scale mental health-care delivery and enable early symptom detection in crisis settings when human resources are overwhelmed. Integrating AI-based triage and monitoring into telepsychiatry platforms could help prioritize high-risk cases, while chatbot assistants might provide immediate psychosocial support or psychoeducation to those awaiting professional care. Early studies underscore improved treatment adherence and symptom outcomes with such tools [54].

## 5. Limitations and Future Directions

Despite its strengths, this study has several limitations that should be acknowledged. First, the sample predominantly consisted of female participants, which introduces a gender imbalance that may affect the generalizability of the findings. Second, the recruitment methods—largely based on digital outreach and online appointment systems—may have favored individuals who were digitally literate and had stable internet access. This likely excluded certain vulnerable subgroups, including older adults, individuals from lower socioeconomic backgrounds, and those with severe psychiatric symptoms or cognitive impairments, who may have been unable to access or engage with telepsychiatry services. These selection biases must be taken into account when interpreting the findings and considering their applicability to the broader population of disaster survivors.

Second, the study primarily focused on PTSD and depressive symptoms, without including structured assessments for anxiety disorders, sleep disturbances, or substance use, which are commonly observed comorbidities in post-disaster contexts. Expanding the diagnostic scope in future studies would yield a more comprehensive understanding of posttrauma psychiatric trajectories.

Third, although the six-month follow-up provides valuable insight into symptom evolution, longer-term functional outcomes (e.g., return to work, social reintegration, quality of life) were not assessed. Future longitudinal studies should incorporate these dimensions to evaluate real-world recovery.

Additionally, variables related to treatment fidelity—such as session attendance, adherence to medication, and dropout rates—were not systematically collected, limiting conclusions on the practical sustainability of telepsychiatric interventions.

Another important limitation is the inability to statistically compare outcomes between patients who received pharmacotherapy in addition to psychoeducation and those who received only psychoeducation. Since this was a naturalistic study, treatment allocation was not randomized or based on clinical judgment. Such comparisons would be confounded by indication: patients not receiving medication were generally more stable at baseline, which might inherently predict better outcomes independently of the intervention type.

A consistent theme in the recent literature is the need to expand the scope of outcomes evaluated in post-disaster mental health research beyond the traditional focus on PTSD and depression. Historically, disaster studies have emphasized PTSD, but anxiety disorders, sleep disturbances, substance misuse, and functional impairments are also prevalent and consequential among survivors [55]. For instance, insomnia is highly prevalent after mass trauma: one study found 38% of adolescents reported persistent sleep problems even 12–24 months after the 2008 Wenchuan earthquake. Increased alcohol and substance use is another documented post-disaster issue, often co-occurring with anxiety and depression and potentially hindering recovery [55] Additionally, disasters can lead to significant functional impairment in daily life: survivors may struggle with work productivity, school performance, or family responsibilities due to ongoing psychological and cognitive effects [56]. Experts now argue that future research and intervention trials should systematically include anxiety, insomnia, substance use, and functional recovery as key outcomes alongside PTSD and depressive symptoms [55,56]. By capturing this broader range of psychosocial consequences, studies can provide a more nuanced understanding of recovery trajectories and treatment needs. In turn, this could inform the development of holistic interventions, for example, integrating sleep restoration programs, substance use counseling, or rehabilitation services into post-disaster mental health care. Emphasizing diverse outcomes will also help identify individuals who, despite not meeting PTSD criteria, still endure serious post-disaster hardships (like chronic insomnia or impaired functioning) and would benefit from targeted support. Ultimately, broadening outcome measures is essential to advance a comprehensive, survivor-centered approach to mental health in disaster contexts, ensuring that interventions address all dimensions of well-being and not just trauma-related syndromes.

Lastly, cultural attitudes toward mental health and digital care were not directly explored, despite their likely impact on service utilization and treatment engagement. Future research should address these social-contextual variables to develop culturally responsive, trauma-informed digital care models.

Future studies should aim to test scalable, evidence-based telepsychiatry protocols through randomized controlled trials while integrating user-centered design to optimize engagement and accessibility—especially in under-resourced populations.

Future studies should also evaluate the applicability and effectiveness of telepsychiatry across various disaster scenarios, including floods, wildfires, and forced displacement. Additionally, research conducted in diverse cultural and geographic contexts is essential to determine the adaptability of remote mental health interventions.

## 6. Conclusions

This study highlights the significant psychological burden following the 6 February 2023 earthquakes in Türkiye, with high rates of PTSD and depression among survivors. Through a telepsychiatry-based intervention, clinically meaningful reductions in PTSD symptoms were observed over a six-month period, supporting the feasibility and effectiveness of digital mental health care in post-disaster settings.

Importantly, the study identified key vulnerability factors—such as female gender, low educational attainment, prior trauma exposure, and limited social support—that should guide targeted screening and early intervention efforts. Telepsychiatry emerged as a promising solution to overcome logistical barriers in disaster zones, but systemic inequities in digital access, service readiness, and national policy frameworks remain critical obstacles to scale.

Moving forward, mental health disaster response strategies must integrate scalable, culturally sensitive, and evidence-based digital care models into national preparedness plans. By addressing both clinical and structural barriers, such models can promote psychological resilience, improve equity, and enhance long-term recovery in vulnerable populations affected by future disasters.

## Figures and Tables

**Table 1:** Sociodemographic characteristics of participants.

Variable	*Mean* ± *SD*/*n* (%)
Age	38.01 ± 13.75
Number of children	1.50 ± 1.63
Gender	
- Female	114 (79.7%)
- Male	29 (20.3%)
Employment status	
- Paid worker	67 (46.9%)
Education	
- Primary school	27 (18.9%)
- High school	34 (23.8%)
- University or above	82 (57.3%)
Married	96 (67.1%)
Region	
- Kahramanmaraş	34 (23.8%)
- Hatay	51 (35.7%)
- Adıyaman	9 (6.3%)
- Others *	49 (34.3%)
Chronic illness	34 (23.8%)

* Others: Gaziantep, Malatya, Kilis, Şanlıurfa, Adana, Osmaniye, Diyarbakır, Elazığ.

**Table 2:** Disaster impact and resource access.

Question/Factor	n (%)
Any personal loss due to the disaster (total)	87 (60.8%)
- Property loss	34 (23.8%)
- Housing loss	74 (51.7%)
- Job/income/education loss	26 (18.2%)
Ability to compensate financial loss (total)	134 (93.7%)
- Personal savings	86 (60.1%)
- Government/social aid	24 (16.8%)
- Family/friends’ support	86 (60.1%)
Limited access to governmental aid	98 (68.5%)
Received psychiatric treatment before the disaster	63 (44.1%)
Sought psychiatric treatment via Psychiatric Association of Türkiye mobile services or local aid organizations	33 (23.1%)
Physical injury	22 (15.4%)
- Minor	16 (11.2%)
- Moderate	4 (2.8%)
- Severe	2 (1.4%)
Hospital admission	
- Outpatient	4 (2.8%)
- Inpatient	2 (1.4%)
Primary caregiver for a dependent	60 (42.0%)
Perceived social support after the earthquake	134 (93.7%)
Volunteered in rescue teams	21 (14.7%)
Trauma before the earthquake	
- Life-threatening danger, injury, domestic violence, torture, witnessing injury, physical violence, sexual assault	14 (9.8%)
- Loss of a loved one, divorce, serious illness, legal issues, job loss, severe poverty	45 (31.5%)
- Two or more from group 1	2 (1.4%)
Feared for own life during the earthquake	117 (81.8%)
Feared for a loved one’s life during the earthquake	123 (86.0%)
Witnessed injury or death of a loved one during the earthquake	44 (30.8%)
Loss of a loved one due to the earthquake *	105 (73.42%)
- Personally witnessed the death of any	40 (27.97%)
- Heard the news from another relative	65 (45.45%)
Able to hold a proper funeral ceremony for the lost one **	
- Proper ceremony	43 (30.1%)
- Improper ceremony	52 (36.4%)
- No body found	48 (33.6%)
Moved to a safe place after the event	
- By my own means/with family support	120 (83.9%)
- With help from others, officials, or rescue teams	23 (16.1%)

* One or more people; ** some of the participants had lost more than one loved ones.

**Table 3:** Categorical diagnosis comparison.

Diagnostic Criterion	1st Month (%)	6th Month (%)
PCL-C > 22	n = 135 (94.4)	n = 117 (81.8)
BDI > 30	n = 111 (77.6)	N/A

**Table 4:** Mean PTSD, depression, and general health scores.

Scale	During the First Month	At the End of the First Month (M ± SD)	At the End of the Sixth Month (M ± SD)	t/*p*
PCL-C Total Score	N/A	42.47 ± 12.22	33.02 ± 12.23	7242/<0.001
PCL-C Re-experiencing	N/A	14.04 ± 4.26	10.31 ± 4.19	7891/<0.001
PCL-C Avoidance	N/A	14.88 ± 5.42	12.38 ± 5.54	4910/<0.001
PCL-C Hyperarousal	N/A	13.60 ± 4.58	10.31 ± 4.47	5769/<0.001
Beck Depression Inventory (BDI)	N/A	36.39 ± 9.69	N/A	N/A
General Health Questionnaire (GHQ-12)	19.34 ± 2.97	N/A	N/A	N/A

**Table 5:** Predictors of PTSD symptom improvement.

Predictor Variable	B Coefficient	Standardized Beta	*p*-Value
Education level	2.081	0.223	0.029
Social support (Yes = 1; No = 2)	−9.001	−0.226	0.027
No past traumatic experience (Yes = 1; No = 2)	5.316	0.223	0.029

**Table 6:** Predictors of chronic PTSD at 6 months.

Predictor Variable	B Coefficient	Standardized Beta	*p*-Value
Beck Depression Inventory	0.472	0.406	0.018
GHQ-12 Score	0.419	0.109	0.529
Age	−0.016	−0.018	0.854
Gender	0.276	0.01	0.919
Social Support	−4.733	−0.112	0.255

**Table 7:** Key characteristics of telepsychiatry studies after major earthquakes.

Disaster (Year, Location)	Study Design and Setting	Population	Telepsychiatry Intervention	Main Findings
Haiti (2010)—Post-earthquake rural clinics	Pilot program with visiting psychiatrist; retrospective chart review. Conducted at 5 rural clinics (Ministry of Health sites) in partnership with a local NGO.	Earthquake survivors in central plateau; N = 65 patients seeking psychiatric services	Hybrid telepsychiatry model: on-site psychiatrist from abroad collaborated with local providers; used mhGAP guidelines. Remote supervision and email consultations for complex cases were available.	Feasible to deliver mental health care in resource-poor, post-disaster settings. Most patients had chronic mental illnesses (depression, epilepsy, psychoses) rather than new trauma-related disorders. Only 5% cited the earthquake as a trigger for their issues, indicating high unmet baseline needs. 90% of patients were kept in care for follow-up, and 69% received psychotropic medications, suggesting telesupervision enabled ongoing treatment.
Wenchuan, China (2008)—Earthquake survivors	Randomized controlled trial (two parallel samples: urban and rural) of a web-based intervention (conducted ~1–2 years post-quake)	Trauma-exposed adults (earthquake survivors); n ≈ 90 in urban sample (internet users) for the RCT (additional rural sample evaluated separately).	Online self-help therapy: Chinese My Trauma Recovery website, an interactive, internet-delivered cognitive–behavioral program for PTSD (in Chinese language). No live therapist; program accessed via computer/mobile over 4 weeks.	Effective in reducing PTSD and distress. Treated survivors showed significantly lower PTSD symptom scores than waiting-list controls. Symptom reduction was rapid (after 1 month of use) and maintained at 3-month follow-up. Large effect sizes reported for improvement in PTSD symptoms demonstrated that unguided digital interventions can reach large numbers of survivors and yield meaningful symptom relief.
Northern Pakistan (2005)—Kashmir earthquake region	Descriptive program report (observational). Telepsychiatry services established in the immediate aftermath and continued during recovery phase (≥1 year)	Populations in remote mountain areas (Azad Jammu and Kashmir and Khyber Pakhtunkhwa) with scarce mental health resources. Included patients with acute trauma reactions and those with preexisting severe mental disorders.	Teleconsultation network: psychiatrists at an urban hub (Rawalpindi) connected via video/telephone with field clinics in disaster zone. Provided remote psychiatric evaluations, treatment guidance, and training of local medics.	Practical and beneficial: telepsychiatry was successfully implemented to bridge specialist care to isolated areas. Enabled prompt psychiatric assessment and management despite infrastructure damage. The program reportedly handled hundreds of consultations, improving referral rates and reducing the need for patient travel. Authors noted telepsychiatry’s feasibility in a disaster context and recommended its integration into national emergency response plans.

## Data Availability

The raw data supporting the conclusions of this article will be made available by the authors on request.

## References

[1] Lechat M.F. (1990). The Public Health Dimensions of Disasters. Int. J. Ment. Health.

[2] Akinci A.C. (2023). 6 Şubat 2023 Kahramanmaraş Depremleri: Sahadan Jeolojik Veriler, Değerlendirme ve Adana Için Etkileri. Cukurova Univ. Muhendis. Fak. Derg..

[3] TTB, SES (2024). Şubat 2023 depremleri 1. Yil Raporu Türk Tabipleri Birliği & Sağlik ve Sosyal Hizmet Emekçileri Sendikasi.

[4] Ministry of Health Türkiye’nin Sağlık Ordusu Deprem Bölgesi İçin Seferber Oldu. https://www.saglik.gov.tr/TR-102172/turkiyenin-saglik-ordusu-deprem-bolgesi-icin-seferber-oldu.html.

[5] World Health Organization (2022). Consolidated Telemedicine Implementation Guide.

[6] Orsolini L., Pompili S., Salvi V., Volpe U. (2021). A Systematic Review on TeleMental Health in Youth Mental Health: Focus on Anxiety, Depression and Obsessive-Compulsive Disorder. Medicina.

[7] Gürcan B.M., Yıldız M. (2021). Online Group Therapy Experience with Schizophrenia Patients During the Pandemic Period. Alpha Psychiatry.

[8] Ministry of Health (2018). Doğum Sonu Bakim Yönetim Rehberi.

[9] Philippe T.J., Sikder N., Jackson A., Koblanski M.E., Liow E., Pilarinos A., Vasarhelyi K. (2022). Digital Health Interventions for Delivery of Mental Health Care: Systematic and Comprehensive Meta-Review. JMIR Ment. Health.

[10] Kılıç C. (1996). General Health Questionnaire: Reliability and Validity Study. Turk. Psychiatry J..

[11] Kocabasoglu N., Yargic I. (2025). The Validity And Safety Of Turkish “PTSD Checklist-Civilian Version” (PCL-C) Scale. New Symp..

[12] Hisli N. (1989). Beck Depresyon Envanterinin Üniversite Öğrencileri Için Geçerliği, Güvenirliği. Psikol. Derg..

[13] Gkintoni E., Vassilopoulos S.P., Nikolaou G. (2025). Next-Generation Cognitive-Behavioral Therapy for Depression: Integrating Digital Tools, Teletherapy, and Personalization for Enhanced Mental Health Outcomes. Medicina.

[14] Zhou X., Kang L., Sun X., Song H., Mao W., Huang X., Zhang Y., Li J. (2013). Prevalence and Risk Factors of Post-Traumatic Stress Disorder among Adult Survivors Six Months after the Wenchuan Earthquake. Compr. Psychiatry.

[15] Farooqui M., Quadri S.A., Suriya S.S., Khan M.A., Ovais M., Sohail Z., Shoaib S., Tohid H., Hassan M. (2017). Posttraumatic Stress Disorder: A Serious Post-Earthquake Complication. Trends Psychiatry Psychother..

[16] Nemeroff C.B., Bremner J.D., Foa E.B., Mayberg H.S., North C.S., Stein M.B. (2006). Posttraumatic Stress Disorder: A State-of-the-Science Review. J. Psychiatr. Res..

[17] Aslan B., Önal Ö. (2024). Prevalence of Probable Post-Traumatic Stress Disorder among Survivors of the 2023 Earthquakes in Türkiye. East. Mediterr. Health J..

[18] Hosseinnejad M., Yazdi-Feyzabadi V., Hajebi A., Bahramnejad A., Baneshi R., Ershad Sarabi R., Okhovati M., Zahedi R., Saberi H., Zolala F. (2022). Prevalence of Posttraumatic Stress Disorder Following the Earthquake in Iran and Pakistan: A Systematic Review and Meta-Analysis. Disaster Med. Public Health Prep..

[19] Cénat J.M., McIntee S.-E., Blais-Rochette C. (2020). Symptoms of Posttraumatic Stress Disorder, Depression, Anxiety and Other Mental Health Problems Following the 2010 Earthquake in Haiti: A Systematic Review and Meta-Analysis. J. Affect. Disord..

[20] Yazawa A., Aida J., Kondo K., Kawachi I. (2022). Gender Differences in Risk of Posttraumatic Stress Symptoms after Disaster among Older People: Differential Exposure or Differential Vulnerability?. J. Affect. Disord..

[21] KOROL M., GREEN B.L., GLESER G.C. (1999). Children’s Responses to a Nuclear Waste Disaster: PTSD Symptoms and Outcome Prediction. J. Am. Acad. Child Adolesc. Psychiatry.

[22] Tang B., Deng Q., Glik D., Dong J., Zhang L. (2017). A Meta-Analysis of Risk Factors for Post-Traumatic Stress Disorder (PTSD) in Adults and Children after Earthquakes. Int. J. Environ. Res. Public Health.

[23] ÇITAK Ş., Dadandı İ. (2024). The Effect of Earthquake Exposure on PTSD Symptoms Is Mediated by Intrusive Rumination and Moderated by Gender: A Cross-Sectional Study on the 2023 Kahramanmaraş Earthquake Survivors. BMC Public Health.

[24] Şam M., Sever G., Yildiz Yüksel H., Aliyev R. (2025). Earthquake Effects on Youth: Understanding Psychological Challenges and Support Needs. BMC Psychiatry.

[25] Hong C., Efferth T. (2016). Systematic Review on Post-Traumatic Stress Disorder Among Survivors of the Wenchuan Earthquake. Trauma Violence Abus..

[26] Sirotich A.C., Camisasca E. (2024). PTSD Risk Factors in Earthquake Survivors and Their Families: A Systematic Review. Eur. J. Psychotraumatology.

[27] D’Angelo M., Valenza M., Iazzolino A.M., Longobardi G., Di Stefano V., Visalli G., Steardo L., Scuderi C., Manchia M., Steardo L. (2024). Exploring the Interplay between Complex Post-Traumatic Stress Disorder and Obsessive–Compulsive Disorder Severity: Implications for Clinical Practice. Medicina.

[28] Gordon-Hollingsworth A.T., Yao N., Chen H., Qian M., Chen S. (2015). Understanding the Impact of Natural Disasters on Psychological Outcomes in Youth from Mainland China: A Meta-Analysis of Risk and Protective Factors for Post-Traumatic Stress Disorder Symptoms. J. Child Adolesc. Trauma.

[29] Başoǧlu M., Kiliç C., Şalcioǧlu E., Livanou M. (2004). Prevalence of Posttraumatic Stress Disorder and Comorbid Depression in Earthquake Survivors in Turkey: An Epidemiological Study. J. Trauma. Stress.

[30] Ying L.H., Wu X.C., Lin C.D., Chen C. (2013). Prevalence and Predictors of Posttraumatic Stress Disorder and Depressive Symptoms among Child Survivors 1 Year Following the Wenchuan Earthquake in China. Eur. Child Adolesc. Psychiatry.

[31] Norris F.H., Friedman M.J., Watson P.J., Byrne C.M., Diaz E., Kaniasty K. (2002). 60,000 Disaster Victims Speak: Part I. An Empirical Review of the Empirical Literature, 1981–2001. Psychiatry Interpers. Biol. Process..

[32] Belen H., Tunca A. (2025). Strength-Based Parenting Improves Depression Outcomes and Promotes Posttraumatic Growth in Earthquake Survivors: A Longitudinal Study. BMC Psychology.

[33] Augusterfer E.F., Mollica R.F., Lavelle J. (2018). Leveraging Technology in Post-Disaster Settings: The Role of Digital Health/Telemental Health. Curr. Psychiatry Rep..

[34] Qadir T.F., Fatima H., Usmani M.H., Hussain S.A. (2016). Telepsychiatry in Pakistan after Natural Disasters. Lancet Psychiatry.

[35] Reinhardt I., Gouzoulis-Mayfrank E., Zielasek J. (2019). Use of Telepsychiatry in Emergency and Crisis Intervention: Current Evidence. Curr. Psychiatry Rep..

[36] Shore J.H., Hilty D.M., Yellowlees P. (2007). Emergency Management Guidelines for Telepsychiatry. Gen. Hosp. Psychiatry.

[37] Ahmed D.R., Heun R. (2024). Standard Guidelines on Electronic Mental Health and Psychosocial Support for Humanitarian Assistance. Lancet Psychiatry.

[38] Grelotti D.J., Lee A.C., Reginald Fils-Aimé J., Solon Jean J., Therosmé T., Petit-Homme H., Oswald C.M., Raviola G., Eustache E., La Jolla M. (2015). A Pilot Initiative to Deliver Community-Based Psychiatric Services in Rural Haiti After the 2010 Earthquake. Ann. Glob. Health.

[39] Sunjaya A.P., Chris A., Novianti D. (2020). Efficacy, Patient-Doctor Relationship, Costs and Benefits of Utilizing Telepsychiatry for the Management of Post-Traumatic Stress Disorder (PTSD): A Systematic Review. Trends Psychiatry Psychother..

[40] Minhas F.A., Nizami A. (2005). Tele-Psychiatry: Answer to Psychiatric Rehabilitation of Earthquake Affected Population in Pakistan. J. Pak. Psychiatr. Soc..

[41] Önder E., Tural Ü., Aker T., Kılıç C., Erdoğan S. (2006). Prevalence of Psychiatric Disorders Three Years after the 1999 Earthquake in Turkey: Marmara Earthquake Survey (MES). Soc. Psychiatry Psychiatr. Epidemiol..

[42] Wang Y., Xu J., Lu Y. (2020). Associations among Trauma Exposure, Post-Traumatic Stress Disorder, and Depression Symptoms in Adolescent Survivors of the 2013 Lushan Earthquake. J. Affect. Disord..

[43] Qi J., Yang X., Tan R., Wu X., Zhou X. (2020). Prevalence and Predictors of Posttraumatic Stress Disorder and Depression among Adolescents over 1 Year after the Jiuzhaigou Earthquake. J. Affect. Disord..

[44] Cénat J.M., Derivois D. (2014). Assessment of Prevalence and Determinants of Posttraumatic Stress Disorder and Depression Symptoms in Adults Survivors of Earthquake in Haiti after 30 Months. J. Affect. Disord..

[45] Altindag A., Ozen S., Sir A. (2005). One-Year Follow-up Study of Posttraumatic Stress Disorder among Earthquake Survivors in Turkey. Compr. Psychiatry.

[46] Guo J., He H., Qu Z., Wang X., Liu C. (2017). Post-Traumatic Stress Disorder and Depression among Adult Survivors 8 Years after the 2008 Wenchuan Earthquake in China. J. Affect. Disord..

[47] Sippel L.M., Liebman R.E., Schäfer S.K., Ennis N., Mattern A.C., Rozek D.C., Monson C.M. (2024). Sources of Social Support and Trauma Recovery: Evidence for Bidirectional Associations from a Recently Trauma-Exposed Community Sample. Behav. Sci..

[48] Diamond P.R., Airdrie J.N., Hiller R., Fraser A., Hiscox L.V., Hamilton-Giachritsis C., Halligan S.L. (2022). Change in Prevalence of Post-Traumatic Stress Disorder in the Two Years Following Trauma: A Meta-Analytic Study. Eur. J. Psychotraumatology.

[49] Morina N., Wicherts J.M., Lobbrecht J., Priebe S. (2014). Remission from Post-Traumatic Stress Disorder in Adults: A Systematic Review and Meta-Analysis of Long Term Outcome Studies. Clin. Psychol. Rev..

[50] Thew G.R., Wild J., Ehlers A. (2023). Early Intervention in Post-traumatic Stress Disorder without Exposure to Trauma Memories Using Internet-delivered Cognitive Therapy: A Pilot Case Series. Br. J. Clin. Psychol..

[51] Hiller R.M., Meiser-Stedman R., Fearon P., Lobo S., McKinnon A., Fraser A., Halligan S.L. (2016). Research Review: Changes in the Prevalence and Symptom Severity of Child Post-traumatic Stress Disorder in the Year Following Trauma—A Meta-analytic Study. J. Child Psychol. Psychiatry.

[52] Gellert G.A., Kabat-Karabon A., Gellert G.L., Rasławska-Socha J., Gorski S., Price T., Kuszczyński K., Marcjasz N., Palczewski M., Jaszczak J. (2024). The Potential of Virtual Triage AI to Improve Early Detection, Care Acuity Alignment, and Emergent Care Referral of Life-Threatening Conditions. Front. Public Health.

[53] Heinz M.V., Mackin D.M., Trudeau B.M., Bhattacharya S., Wang Y., Banta H.A., Jewett A.D., Salzhauer A.J., Griffin T.Z., Jacobson N.C. (2025). Randomized Trial of a Generative AI Chatbot for Mental Health Treatment. Nejm AI.

[54] Gutierrez G., Stephenson C., Eadie J., Asadpour K., Alavi N. (2024). Examining the Role of AI Technology in Online Mental Healthcare: Opportunities, Challenges, and Implications, a Mixed-Methods Review. Front. Psychiatry.

[55] Morganstein J.C., Ursano R.J. (2020). Ecological Disasters and Mental Health: Causes, Consequences, and Interventions. Front. Psychiatry.

[56] Makwana N. (2019). Disaster and Its Impact on Mental Health: A Narrative Review. J. Fam. Med. Prim. Care.

